# VCAM-1 Is Upregulated in Uranium Miners Compared to Other Miners

**DOI:** 10.3390/life11111223

**Published:** 2021-11-11

**Authors:** Nour A. Ass’ad, Xin Shore, Orrin Myers, Alexandra R. Camacho, Quiteria Jacquez, Charles Pollard, Linda S. Cook, Shuguang Leng, Kimberly Page, Akshay Sood, Katherine E. Zychowski

**Affiliations:** 1 Department of Internal Medicine, University of New Mexico School of Medicine, Albuquerque, NM 87131, USA; nassad@salud.unm.edu (N.A.A.); lcook@salud.unm.edu (L.S.C.); SLeng@salud.unm.edu (S.L.); pagek@salud.unm.edu (K.P.); ASood@salud.unm.edu (A.S.); 2 Department of Family and Community Medicine, University of New Mexico School of Medicine, Albuquerque, NM 87131, USA; xinwang1@salud.unm.edu (X.S.); Omyers@salud.unm.edu (O.M.); 3 College of Nursing, University of New Mexico-Health Sciences Center, Albuquerque, NM 87131, USA; ARCamacho@salud.unm.edu (A.R.C.); qsanchez@salud.unm.edu (Q.J.); 4 Miners’ Colfax Medical Center, 203 Hospital Drive, Raton, NM 87740, USA; cpollard@minershosp.com; 5 Department of Epidemiology, School of Public Health, University of Colorado-Anschutz, Arora, CO 80045, USA

**Keywords:** VCAM-1, inflammation, cardiovascular, mining

## Abstract

The United States has a rich history of mining including uranium (U)-mining, coal mining, and other metal mining. Cardiovascular diseases (CVD) are largely understudied in miners and recent literature suggests that when compared to non-U miners, U-miners are more likely to report CVD. However, the molecular basis for this phenomenon is currently unknown. In this pilot study, a New Mexico (NM)-based occupational cohort of current and former miners (*n* = 44) were recruited via a mobile screening clinic for miners. Serum- and endothelial-based endpoints were used to assess circulating inflammatory potential relevant to CVD. Non-U miners reported significantly fewer pack years of smoking than U-miners. Circulating biomarkers of interest revealed that U-miners had significantly greater serum amyloid A (SAA), soluble intercellular adhesion molecule 1 (ICAM-1, ng/mL), soluble vascular cell adhesion molecule 1 (VCAM-1, ng/mL), and VCAM-1 mRNA expression, as determined by the serum cumulative inflammatory potential (SCIP) assay, an endothelial-based assay. Even after adjusting for various covariates, including age, multivariable analysis determined that U-miners had significantly upregulated VCAM-1 mRNA. In conclusion, VCAM-1 may be an important biomarker and possible contributor of CVD in U-miners. Further research to explore this mechanism may be warranted.

## 1. Introduction

Mining demonstrates significant risks for long-term health effects. Chronic health conditions such as lung diseases [1,2,3] and to a lesser extent, cardiovascular diseases (CVD), may impact uranium (U) miners [4,5,6]. The Southwestern U.S. has a rich culture of mining, including coal, U, and other metals. The majority of U-mines cumulatively produced more than 225 million tons of ore between 1949–1989 [7], with some current active U-mining in the western states. Mining and affiliated professions such as processing and transporting U-ore have been investigated with respect to CVD, but results are conflicting. Recently, U-miners exhibited an increase in self-reported angina compared to non-U miners, despite having lower body mass index (BMI), lower nicotine use, and lower duration of mining tenure than non-U miners [8]. In a study of French U-miners, CVD death rates were higher among U-miners, with a significantly higher risk of cerebrovascular mortality [9,10]. In contrast, other cohort studies have determined no evidence in U-miner mortality from CVD [4]. In a French U-miners’ cohort, authors observed that specific exposure to soluble, reprocessed U may increase CVD mortality [11].

Endothelial dysfunction is considered to be a sentinel physiological indicator of subsequent CVD [12]. Endothelial cells express adhesion molecules upon systemic insult and VCAM-1 is an endothelial adhesion molecule, upregulated upon cellular activation [13]. VCAM-1 and other markers may be predictive of CVD risk in both men and women [14]. This adhesion molecule promotes binding to circulating leukocytes via very late antigen-4 (VLA-4), and promotes leucocyte adhesion and transendothelial migration, which subsequently contribute to atherosclerotic plaque development [15]. Our lab and others have demonstrated that endothelial responses to patient serum can be used to assess circulating inflammatory potential, relevant to CVD, even in the presence or absence of canonical inflammatory biomarkers, such as cytokines [16,17,18]. This novel endothelial bioassay, the ‘serum cumulative inflammatory potential assay’ (SCIP assay), has pinpointed circulating factors as drivers of CVD, especially following inhaled exposures or other lung injury-inducing diseases. Other inflammatory markers such as interleukins (such as IL-8) and serum amyloid A (SAA) have also demonstrated a role in driving CVD. For example, one study found that SAA was a predictor of coronary artery disease (CAD) and CVD in women [19]. Another study determined the IL-8 signaling directly augments endothelial cell survival, proliferation, and matrix metalloproteinase production via regulation of angiogenic pathways [20].

The aim of this pilot clinical study was to assess molecular biomarkers of CVD, including implementing a novel endothelial bioassay [21,22], in a cohort of miners. We postulated that U-miners would exhibit a significantly more inflammatory and pathological phenotype than non-U miners, based on circulating molecular and endothelial endpoints. To our knowledge, this is the first assessment of molecular CVD biomarkers in a mining occupational cohort. This inaugural study is of critical importance to understanding occupational exposures in hazardous industries, such as mining, and long-term health outcomes.

## 2. Materials and Methods

### 2.1. Patient Recruitment and Biospecimen Collection

**Figure 1:**
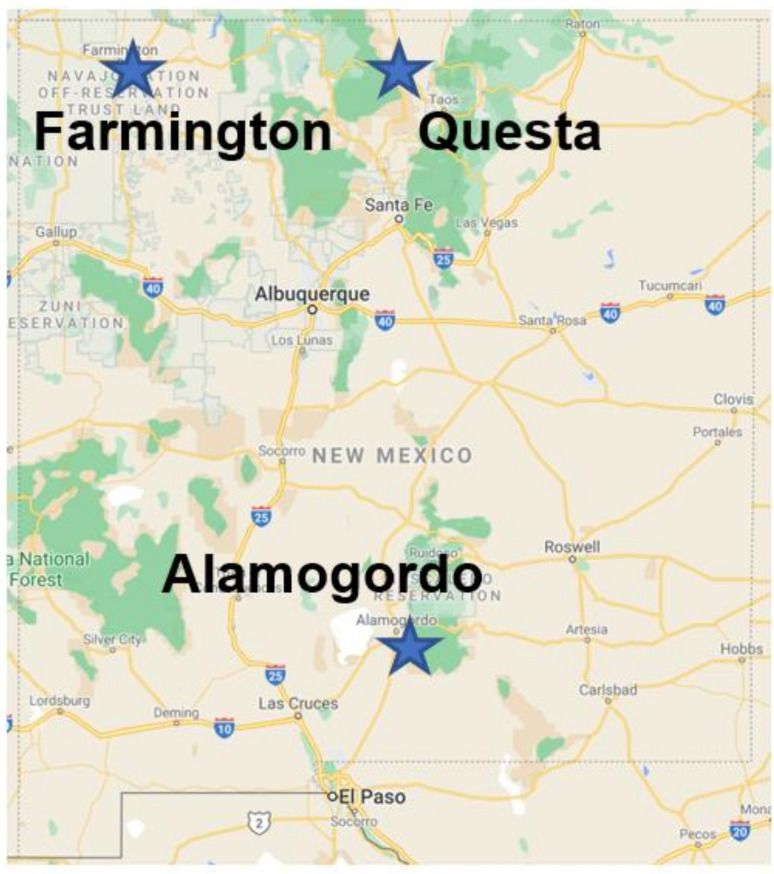
A rural mobile screening clinic through Miners’ Colfax Medical Center was held in Questa, Alamogordo, and Farmington, NM, periodically to recruit 44 patient participants.

This cross-sectional study was a joint project between Miners’ Colfax Medical Center (MCMC) and the University of New Mexico Health Sciences Center. All patient subjects were recruited from the MCMC mobile outreach. The MCMC mobile screening clinic is held once a month at rural locales including Questa, Farmington, and Alamogordo, NM, USA (Figure 1). This screening clinic consists of a 53 ft long platform with a generator for the power supply. Outreach clinics were advertised to communities via print, media, radio, as well as through community/church leaders, in addition to self-referral. Patients completed a screening survey, underwent a routine physical exam, and were assessed for respiratory symptoms of disease. Patients completed a clinical health survey to address CVD endpoints including self-reported medical history. Blood was drawn immediately following the clinical visit and extracted serum was flash frozen for further analyses through the UNM Molecular Epidemiology Laboratory. Risk factors for CVD, assessed at the time of examination included age, sex, race/ethnicity, body mass index, smoking status, self-reported hypertension, measured systolic or diastolic hypertension, and medication history. In addition, a basic blood panel including total cholesterol, HDL, LDL, triglyceride levels and markers of insulin resistance including hemoglobin A1c (HbA1c), non-fasting glucose, and insulin levels were evaluated in each participant. A total of 44 patient subjects were recruited for the purposes of this study (10 U-miners and 34 non-U miners).

### 2.2. Inclusion Criteria and Mining Status

Recruited subjects were required to be employed in the mining industry at least 1 year and all participants provided total years of mining tenure. Exposure status was defined as those who were ever employed in the U-mining industry (U-miners) vs. those who mined other ores including coal, metal, and non-metals (non-U miners).

### 2.3. Serum Cumulative Inflammatory Potential (SCIP) Assay

Human coronary artery endothelial cells (hCAECs, Lonza) were grown in confluence T75 sterile flasks (hCAECs, Lonza). Basal complete media were replaced every other day and cells were passaged once per week. Human coronary artery endothelial cells were seeded in a 24-well plate and grown to confluence in complete media. For the 44 deidentified patient serum samples (with no name, medical record number, or birthday), 25 μL serum in 475 μL of serum-free commercial media, 5% serum by volume per well, were incubated with a confluent hCAEC monolayer. Samples were incubated at 37 °C for 4 h. Total RNA was extracted from cells using a commercially-available kit (RNeasy, Qiagen, Germantown, Maryland) based on the manufacturer’s instructions. The extracted RNA was then aliquoted in cryosafe centrifuge tubes and frozen at −80 °C. RNA was then thawed and reverse transcribed using High-Capacity cDNA Reverse Transcription Kits (Applied Biosystems, Foster City, CA, USA), prior to qPCR. Using the Taqman Gene Expression protocol, gene expression was assessed including VCAM-1 (Hs01003372_m1), ICAM-1 (Hs01003372_m1), and chemokine ligand-2 (CCL-2, Hs00234140_m1), as per the manufacturer’s instructions (ThermoScientific, Waltham, MA, USA). Target genes were normalized to TATA binding protein (TBP, Hs99999910_m1). Relative gene expression using endothelial targets were analyzed using the 2^−∆∆CT^ method, as previously described [23]. Endothelial transcriptional responses to serum was evaluated using qPCR. Genes evaluated included chemokine ligand 2 (CCL-2), ICAM-1, and VCAM-1.

### 2.4. Serum Cytokine Analysis

The V-PLEX vascular injury panel 2 (human) kit (Mesoscale Discovery, Rockville, MD, USA) measures 4 serum-borne biomarkers, critical to acute inflammation and tissue damage in vascular disease. This is an electrochemiluminescence (ECL) detection technique that differs somewhat from traditional colormetric, antibody-based ELISAs. These included serum amyloid A (SAA), C-reactive protein (CRP), VCAM-1, and ICAM-1. This protocol was performed using the appropriate serum volume per biological sample, according to the manufacturers’ instructions. Additional circulating cytokines assessed included interferon gamma (IFN-γ), interleukin-10 (IL-10), interleukin-6 (IL-6), interleukin-8 (IL-8), and tumor necrosis factor alpha (TNF-α).

### 2.5. Statistical Analysis

We tested whether exposure to uranium mining was associated with differences in inflammation biomarker levels and with other miner characteristics. Inflammation biomarkers are described above, and miner characteristics included gender, diabetes, age, systolic blood pressure (SBP), diastolic blood pressure (DBP), mean arterial pressure (MAP) cholesterol, low density lipoprotein (LDL), years of mining (mining tenure), pack years, HbA1c, statins, insulin, and glucose. Unadjusted associations between uranium miner status and continuous variables were tested using Student’s *t*-tests and non-parametric Wilcoxon tests. Multivariable linear model variable selection analyses were conducted for each inflammation biomarker with uranium miner status forced into the model and adding other potential confounders as covariates. Due to the small sample size. no more than 2 additional parameters were added into models. Dependent variables were log-transformed before analyses. Fit of models was summarized using the adjusted coefficient of determination (R-squared) and Akaike’s Information Criteria corrected for small samples (AICC). For AICC, smaller values indicate a more favorable tradeoff between the number of parameters and fit, and models that differ by less than 2.0 AICC units (∆AICC ≤ 2) are essentially equivalent models [24]. Data were analyzed using Statistical Analysis Software 9.4 (SAS Institute Inc., Cary, NC, USA, 2020).

## 3. Results

### 3.1. Demographics and Baseline Health Metrics

Demographic and patient characteristics relevant to CVD are presented in Table 1 between U and non-U miners. The majority of participants were male (80%), similar to previous studies of miners [8,25]. The majority of participants were Hispanic (61%), followed by White (25%) and American Indian (14%) (Table 1). Uranium miners were significantly older than non-U miners (*p* = 0.04) and had significantly more pack years of smoking (*p* < 0.01) (Table 2). Furthermore, U-miners weighed significantly more than non-U miners (*p* = 0.02); however, BMI did not demonstrate any significant difference. Measured blood pressure (diastolic, systolic, mean arterial), mining tenure, cholesterol, HDL, LDL, triglycerides, HbA1c, glucose, and insulin did not significantly differ between U-miners and non-U miners.

### 3.2. Continuous Molecular Biomarkers

Univariate analysis indicated several detected biomarkers of CVD were upregulated in U-miners, compared to non-U miners including serum amyloid A (SAA) (*p* = 0.049), soluble ICAM-1 (*p* = 0.04), VCAM-1 (*p* = 0.03), IL-8 (*p* = 0.01), and VCAM-1 as measured in the SCIP assay (*p* < 0.01) (Table 3).

### 3.3. Multivariable Models

Multivariable models, adjusted for individual covariates revealed several significantly upregulated continuous molecular biomarkers (Table 4). The VCAM-1 mRNA (SCIP assay) indicated significant association with U-mining status, even when adjusting for age and hdl (adjusted R^2^ = 0.29, *p* = 0.034), the model with the smallest AICC, ∆AICC = 0. Models that adjusted only for age and for diabetes plus age had less support based on ∆AICC with the latter model also having a weaker association between U-mining status and VCAM-1. The U-mining coefficient for the diabetes plus hdl model was 0.05 units lower then best-fitting model and was not different from zero (*p* = 0.088). Serum amyloid A (SAA, ng/mL), IL-8 (pg/mL), and soluble VCAM-1 (ng/mL) also demonstrated significant association in the U-miners group, even when adjusting for covariates (Table 4). For these biomarkers and models with ∆AICC < 2.0, there is little evidence of substantial confounding of the U-mining status association. Other tested biomarkers did not demonstrate statistical significance using either univariate or multivariable modeling.

## 4. Discussion

### 4.1. Key Features of These Findings

This cross-sectional study indicates that U-mining is associated with upregulation of soluble VCAM-1 and VCAM-1 mRNA expression, as measured by the SCIP assay, even after adjusting for individual, confounding covariates such as age, diabetes, HbA1c, or statin usage. U and other metals’ exposure are key contributors to the development of chronic vascular diseases and vascular events [26,27]. In this pilot study, we found that U-miners exhibited both upregulated cell adhesion molecules such as ICAM-1 and VCAM-1, which mediate leukocyte recruitment into organs following radiation exposure [28].

Upregulation of these individual adhesion molecules has been implicated in both endothelial dysfunction and increased risk of CVD [29]. Studies have found that SAA and IL-8 have also been implicated in the development of atherosclerosis [30,31]. There is conflicting literature regarding the association between CVD risk and radon or external gamma radiation exposure [32], which may suggest that other mechanisms such as mixed-metals’ exposure as the culprit driving CVD in miners. Other studies have noted an increase in inflammatory potential, as measured by the SCIP assay in Navajo residents living in close proximity to abandoned U-mines [17,26]. Our approach of using a novel, remote clinical screening platform to assess molecular biomarkers linked with CVD ultimately demonstrates the utility of remote screening to evaluate CVD risk. Future studies may focus on basic inflammatory mechanisms that may be driving upregulation of these biomarkers.

### 4.2. Strengths of This Study

Strengths of this study include the study population that represents underserved, minority populations including White, Native American, and Hispanic miners. The mobile, rural recruitment strategy also allowed for broader geographic inclusion, including reaching individuals who are less likely to attend a hospital-based, fixed clinic. In addition, assessment of molecular biomarkers in an at-risk, occupational cohort is novel and important to understanding possible molecular targets relevant to occupational and toxicological exposures.

### 4.3. Potential Limitations

The major limitation of this study was the small sample size, which decreases statistical power and increases uncertainty in selecting the best model. We are aware that these results should be repeated with a greater number for both U and non-U miners, and ongoing studies are in the process of recruiting more patients from multiple sites. Future studies will include miners recruited from both MCMC mobile outreach and the UNM-H Pulmonology Clinic. Additionally, U-mining tenure (years of U-mining) was based on self-report and therefore subjected to recall bias. Furthermore, although the SCIP assay is sensitive, detecting endothelial dysfunction in lieu of canonical cytokine changes, it is not necessarily specific to occupational exposures. However, given the preliminary and pilot nature of this study, the data collected from our study are immensely valuable.

## 5. Conclusions

These findings suggest that U-miners may be at higher risk for CVD, based on their molecular biomarkers and that the SCIP assay may serve as a novel way to assess circulating inflammatory potential following occupational exposures in the mining industry. Further research is warranted to decipher specific circulating, serum-borne factors that may play a role in occupational disease.

## Figures and Tables

**Table 1:** Characteristics of study participants.

	All (*n* = 44)		Non-U Miners (*n* = 34)		U-Miners (*n* = 10)	
	*n*	(%)	*n*	(%)	*n*	(%)
**Gender**						
Male	42	(95)	34	(100)	8	(80)
Female	2	(5)	0	0	2	(20)
**Ethnicity**						
White	11	(25)	9	(26)	2	(20)
Hispanic	27	(61)	19	(56)	8	(80)
American Indian	6	(14)	6	(18)	0	0
**Current Smoker**						
Yes	5	(11)	4	(12)	1	(10)
No	39	(89)	30	(88)	9	(90)
**Diabetic**						
Yes	8	(18)	4	(12)	4	(40)
No	36	(82)	30	(88)	6	(60)
**Hypertensive**						
Yes	16	(36)	13	(38)	3	(30)
No	28	(64)	21	(62)	7	(70)
**High Cholesterol**						
Yes	10	(23)	8	(24)	2	(20)
No	34	(77)	26	(76)	8	(80)
**Coronary artery disease**						
No	44	(100)	34	(100)	10	(100)
**Statin usage**						
Yes	7	(16)	5	(15)	2	(20)
No	28	(64)	20	(59)	8	(80)
Unknown	9	(20)	9	(26)	0	0
**HDL Value**						
HDL < 40	17	(39)	15	(44)	2	(20)
HDL 40+	27	(61)	19	(56)	8	(80)
**LDL Value**						
No data	1	(2)	0	0	1	(10)
LDL ≤ 100	30	(68)	23	(68)	7	(70)
LDL > 100	13	(30)	11	(32)	2	(20)
**Triglycerides Value**						
Trig < 150	21	(48)	16	(47)	5	(50)
Trig 150+	23	(52)	18	(53)	5	(50)
**Glucose**						
60–139 gm/dL: normal	40	(91)	30	(88)	10	(100)
140–199 mg/dL: at risk	2	(5)	2	(6)	0	0
200+ mg/dL	2	(5)	2	(6)	0	0
**HbA1c**						
HbA1c ≤ 7	38	(86)	29	(85)	9	(90)
HbA1c > 7	6	(14)	5	(15)	1	(10)
**Body Mass Index**						
BMI < 25	6	(14)	4	(12)	2	(20)
BMI ≥ 25	38	(86)	30	(88)	8	(80)

**Table 2:** Characteristics of study participants based on mining type.

	Non-U Miners		U-Miners		
	Mean	Std	Mean	Std	*p*-Value ^a^
Age	55.9	16.2	66.9	6.2	**0.04**
Pack-years	7.5	11.3	24.3	16.7	**<0.01**
Years of mining exposure	20.0	13.9	16.8	13.6	0.63
Years of uranium exposure	0	0	4.4	3.9	N/A
BMI	32.1	6.8	27.5	4.3	0.07
Systolic Blood Pressure	131.6	18.1	133.4	18.2	0.68
Diastolic Blood Pressure	80.7	7.7	84.0	6.6	0.15
Mean Arterial Pressure, mm Hg	97.7	10.3	100.4	10.3	0.43
Cholesterol Value	161.5	35.6	151.3	39.0	0.50
HDL Value	41.6	9.4	48.3	14.4	0.10
LDL Value	86.7	35.6	77.8	39.2	0.54
Triglycerides Value:	190.6	116.3	182.3	133.0	0.57
HbA1c	6.4	2.0	6.0	1.0	0.99
Glucose	111.4	67.3	101.9	18.3	0.57
Insulin	29.3	31.3	35.9	64.3	0.54

^a^ Nonparametric Wilcoxon Rank Sum Test.

**Table 3:** Comparison of Circulating Inflammatory Molecular Biomarkers and Inflammatory Potential.

	Non-U Miners		U-Miners		
	Mean	Std	Mean	Std	*p*-Value ^a^
CRP (ng/mL)	3085	3287	6428	7002	0.14
SAA(ng/mL)	2606	3234	11193	22179	**0.049**
soluble ICAM-1 (ng/mL)	359.9	93.3	449.2	128.0	**0.04**
soluble VCAM-1 (ng/mL)	377.9	115.1	540.3	246.6	**0.03**
IFN-γ (pg/mL)	6.91	4.36	8.74	3.79	0.11
IL-10 (pg/mL)	0.31	0.12	0.34	0.15	0.61
IL-6 (pg/mL)	2.30	3.00	3.30	4.20	0.42
IL-8 (pg/mL)	19.80	8.67	31.14	15.27	**0.01**
TNF-α (pg/mL)	4.10	1.17	4.57	0.82	0.30
SCIP assay-CCL-2 mRNA expression	1.00	0.51	1.21	0.62	0.27
SCIP assay-VCAM-1 mRNA expression	1.00	0.68	1.75	0.68	**<0.01**
SCIP assay- ICAM-1 mRNA expression	1.00	0.22	1.04	0.15	0.39

^a^ Nonparametric Wilcoxon Rank Sum Test.

**Table 4:** Multivariable linear models for association between inflammation biomarkers and U-mining status.

		U-Miner Coefficient				
Inflammation Biomarker	Model Covariates	U-Mining β Coefficient Estimate	Standard Error	*p*-Value	Model Adjusted R-Squared	∆AICC
VCAM-1 mRNA (SCIP assay)	U-mining+age	0.33	0.14	0.015	0.26	0.43
	U-mining+diabetes	0.39	0.15	0.007	0.17	5.56
	U-mining+hdl	0.36	0.14	0.010	0.23	2.13
	U-mining+pack years	0.39	0.16	0.016	0.15	6.27
	U-mining+age+hdl	0.29	0.14	0.034	0.29	0.00
	U-mining+age+systolic blood pressure	0.34	0.14	0.014	0.24	2.85
	U-mining+diabetes+hdl	0.24	0.14	0.088	0.28	0.69
SAA (ng/mL)	U-mining+systolic blood pressure	0.91	0.38	0.016	0.15	0.00
	U-mining+mean arterial pressure	0.87	0.38	0.022	0.13	0.77
	U-mining+pack years	1.21	0.43	0.005	0.13	1.33
	U-mining+pack years+hdl	1.21	0.44	0.012	0.11	3.33
	U-mining+pack years+systolic blood pressure	1.20	0.17	0.004	0.17	0.45
VCAM-1 (ng/mL)	U-mining+age	0.29	0.12	0.014	0.13	2.89
	U-mining+pack years	0.29	0.13	0.026	0.13	3.11
	U-mining+hba1c	0.33	0.11	0.003	0.18	0.70
	U-mining+statin usage	0.26	0.11	0.021	0.19	0.00
	U-mining+statin usage+ hba1c	0.28	0.11	0.010	0.22	0.04
IL-8 (pg/mL)	U-mining+diastolic blood pressure	0.40	0.15	0.008	0.15	2.04
	U-mining+mean arterial pressure	0.41	0.15	0.006	0.14	2.10
	U-mining+cholesterol	0.40	0.14	0.005	0.19	0.00
	U-mining+cholesterol+diastolic blood pressure	0.37	0.15	0.011	0.18	1.97

## Data Availability

Data presented in this study are available upon request from the corresponding author.
